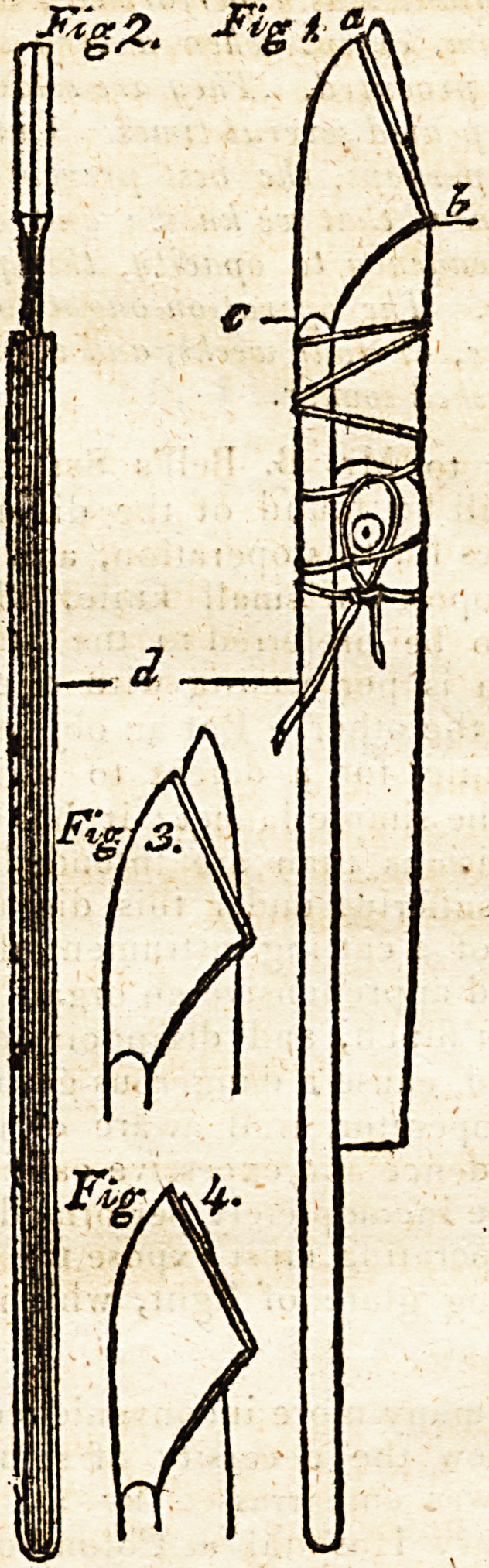# Mr. Leath's New Lancet

**Published:** 1809-07

**Authors:** J. G. Leath

**Affiliations:** Assistant Surgeon of the 19th Regt. of Foot


### *To the Editors of the Medical and Physical Journal.*

GENTLEMEN,

THE general utility, and sometimes necessity, of making frequent divisions of the inflamed vessels in cases of Ophthalmia, is admitted by all who have written and practised on that disease; but the operation, by the means hitherto in use, is attended with so much difficulty and danger, that it is often omitted when it would not fail to administer great relief. The following citation, from a high authority, is a sufficient sanction for this remark.

*These operations require great nicety. For the particular mode of performing them the reader is referred to the writers*

*writers on Surgery. Much harm will ensue from them, when they are injudiciously performed; and they ought, to be refrained from, except when a very skilful and expert surgeon can be procured. They are seldom serviceable, except they be repeated several times. Cutting the vessels of the adnata is, perhaps, the best preventative against opacity of the cornea, that we know; and whenever there appears the least tendency to opacity, this practice should be put in execution. The operation ought to be repeated daily for two, or three, or four weeks, and even longer, if a cure be not accomplished sooner.*

By referring to Mr. B. Bell's System of Surgery, an enumeration will be found of the different modes in use at different times for this operation, and his objections to them. He proposes a small knife, which, having one edge only, is to be preferred to the lancet; that, while the scarification is performing with one edge, endangers the eyelid with the other. But an objection may be made even to the knife, for a defect to which it is liable in common with the simple lancet; it has no guard, to prevent deeper incisions than are intended: and the irritability of those suffering under this disease, joined to the natural horror of a cutting instrument about to be applied to so tender and apprehensive an organ as the eye, may well make them flinch, and disappointing the most sure and expert hand, cause a dangerous gashing of the wrong part. If the operator, well aware of this danger, proceeds with diffidence and excessive caution, the operation may not only be incompletely performed at last, but such a manner of operating must expose the patient for a long time to a strong glare of light, which is intolerable in this disease.

I could state many more inconveniences; but these are sufficient to shew the necessity of some better mode of operation. I was embarrassed by all these difficulties with the Military Hospital at Colombo, full of this disease. I made several attempts, and at last succeeded in adapting the lancet by a guard, to perform effectually this operation free from all hazard.

I trust that the following drawing and description will give an adequate idea of the instrument.

Fig.

Fig. 1. Represents a side view of the instrument with the lancet fixed.

*a b c* Two small triangular plates united at the back from *a* to *c*, and separated at the front, to admit the blade of a lancet; from *a* to *b* are the edges, which are made thick, and form shoulders to the edge of the lancet; the other parts of the plate are as light as they can be made without substracting from due strength; and the plates are joined to a common director, which forms a handle handle to them, and which is grooved, to receive the handle of the lancet.

Fig. 2. Gives a front view of the instrument.

Fig. 4. The proportion of the blade that I expose for making the scarification on the eye.

Fig. 3. The proportion of blade for making incisions in the tonsils; and fig. 1. for opening the sublingual vein, in both which operations, the lancet thus guarded and limited, will be found eminently useful; and, indeed, wherever great nicety of operation is required. By cutting off part of the handle as at letter *d*, it remains as effective for making the scarifications on the eye, and may then be carried in the lancet case an inseparable companion, (as it ought to be, and I trust will be, when it is well known through the medium of your Journal) to the lancet: I, however, retain it the full length, because I find it so useful in operations of many kinds, where a greater length of handle is required than for scarification of the eye.

With the lancet thus guarded, it is evident that a youth, who has only learned to bleed, may perform with safety and effectualness this important, and hitherto difficult and dangerous operation.

I am, &c.

J. G. LEATH,

Assistant Surgeon of the 19th Regt. of Foot.

## Figures and Tables

**Fig 1. Fig 2. Fig 3. Fig 4. f1:**